# Anti-adipogenic effects of KD025 (SLx-2119), a ROCK2-specific inhibitor, in 3T3-L1 cells

**DOI:** 10.1038/s41598-018-20821-3

**Published:** 2018-02-06

**Authors:** Duy Trong Vien Diep, Kyungki Hong, Triyeng Khun, Mei Zheng, Asad ul-Haq, Hee-Sook Jun, Young-Bum Kim, Kwang-Hoon Chun

**Affiliations:** 1 Gachon Institute of Pharmaceutical Sciences, College of Pharmacy, Gachon University, Incheon, 21936 Republic of Korea; 2 Division of Endocrinology, Diabetes and Metabolism, Beth Israel Deaconess Medical Center and Harvard Medical School, Boston, Massachusetts, United States; 3 Lee Gil Ya Cancer and Diabetes Institute, Gachon University, Incheon, 21999 Republic of Korea; 4 Gachon Medical Research Institute, Gil Hospital, Incheon, 21565 Republic of Korea

## Abstract

Adipose tissue is a specialized organ that synthesizes and stores fat. During adipogenesis, Rho and Rho-associated kinase (ROCK) 2 are inactivated, which enhances the expression of pro-adipogenic genes and induces the loss of actin stress fibers. Furthermore, pan ROCK inhibitors enhance adipogenesis in 3T3-L1 cells. Here, we show that KD025 (formerly known as SLx-2119), a ROCK2-specific inhibitor, suppresses adipogenesis in 3T3-L1 cells partially through a ROCK2-independent mechanism. KD025 downregulated the expression of key adipogenic transcription factors PPARγ and C/EBPα during adipogenesis in addition to lipogenic factors FABP4 and Glut4. Interestingly, adipogenesis was blocked by KD025 during days 1~3 of differentiation; after differentiation terminated, lipid accumulation was unaffected. Clonal expansion occurred normally in KD025-treated cells. These results suggest that KD025 could function during the intermediate stage after clonal expansion. Data from depletion of ROCKs showed that KD025 suppressed cell differentiation partially independent of ROCK’s activity. Furthermore, no further loss of actin stress fibers emerged in KD025-treated cells during and after differentiation compared to control cells. These results indicate that in contrast to the pro-adipogenic effect of pan-inhibitors, KD025 suppresses adipogenesis in 3T3-L1 cells by regulating key pro-adipogenic factors. This outcome further implies that KD025 could be a potential anti-adipogenic/obesity agent.

## Introduction

Fats, or triacylglycerols, are highly efficient sources of energy in the body, and mammals have developed intricate mechanisms to store fats in adipocytes to minimize the loss of energy. Adipose tissue is a hormone-secreting organ that plays an important role in maintaining organism homeostasis. The role of adipocytes is gaining more interest because its dysfunction is considered a major cause of obesity, type 2 diabetes, and various metabolic diseases^1^. Generally, adipogenesis occurs in two phases: the determination phase, involving the conversion of mesenchymal stem cells (MSCs) to the adipocyte lineage or pre-adipocytes, and the terminal differentiation phase in which pre-adipocytes develop into mature adipocytes^2^.

During terminal differentiation, key transcription factors are expressed sequentially and elaborately, such as the peroxisome proliferator-activated receptor γ (PPARγ), nuclear receptor, and CCAAT-enhancer-binding protein (C/EBP) transcription factors. In particular, PPARγ is a member of the nuclear-receptor superfamily and has been considered the master regulator in adipogenesis. PPARγ is not only required, but also sufficient, for adipogenesis and for the maintenance of adipocyte characteristics^3–6^. PPARγ and C/EBPα induce the expression of various metabolic genes that are required to maintain adipocyte phenotypes, such as fatty acid-binding protein 4 (FABP4; aP2) and glucose transporter 4 (GLUT4; SLC2A4)^2^. PPARγ and C/EBPα expression are induced by C/EBPβ and C/EBPδ, early transcription factors activated within hours of an adipogenic stimuli^1^.

Rho-associated coiled-coil-containing protein kinases (ROCKs) were first introduced as RhoA-binding proteins that regulate actin cytoskeleton remodeling in cells^7,8^. ROCK1 (ROKβ) and ROCK2 (ROKα) have high similarity in the amino and carboxyl termini, which contain the catalytic kinase domain and the Rho-binding domain (RBD), respectively, whereas they exhibit relatively low homology in the coiled-coil region, with only 55% identity. ROCK isoforms play pivotal roles in the regulation of actin cytoskeleton organization, cytokinesis, differentiation, apoptosis, glucose metabolism, cell adhesion/motility, and inflammation^2,9–11^. The Rho GTPase-Rho-associated kinase (ROCK) signaling pathway inhibits adipocyte differentiation and can be explained by several plausible mechanisms. Rounded cell morphology and loss of stress fibers are prerequisites for adipocyte differentiation^2,12–14^, during which Rho GTPase and ROCK activity must be suppressed^15,16^. In addition, active Rho promotes the expression of YAP (Yes-associated protein) and TAZ (transcriptional co-activator with PDZ-binding motif), transcription factors that suppress adipocyte differentiation^17^. The decrease in adiposity of p190B-RhoGAP-deficient mice further supports these findings^18^. One study, based on knockdown and genetic approaches, demonstrated that only the ROCK2 isoform has anti-adipogenic functions in 3T3-L1 and mouse embryonic fibroblasts (MEFs)^19^. Y-27632, a pan-inhibitor of ROCK1 and 2, promoted adipocyte differentiation in 3T3-L1 cells and exhibited similar function to insulin in this study. However, the exact role and mechanism of ROCK2 in adipogenesis needs to be elucidated in greater detail.

In the present study, we investigated the ROCK2-specific function in adipogenesis using a ROCK2-specific inhibitor, KD025 (formerly known as SLx-2119)^10^. We provide important evidence that KD025 suppresses adipocyte differentiation in 3T3-L1 cells by inhibiting the expression of pro-adipogenic factors such as PPARγ and C/EBPα. We suggest that KD025 could suppress adipogenesis by targeting an unknown adipogenic factor other than ROCK2.

## Results

### Effect of KD025 on differentiation of 3T3-L1 adipocytes

**Figure 1:**
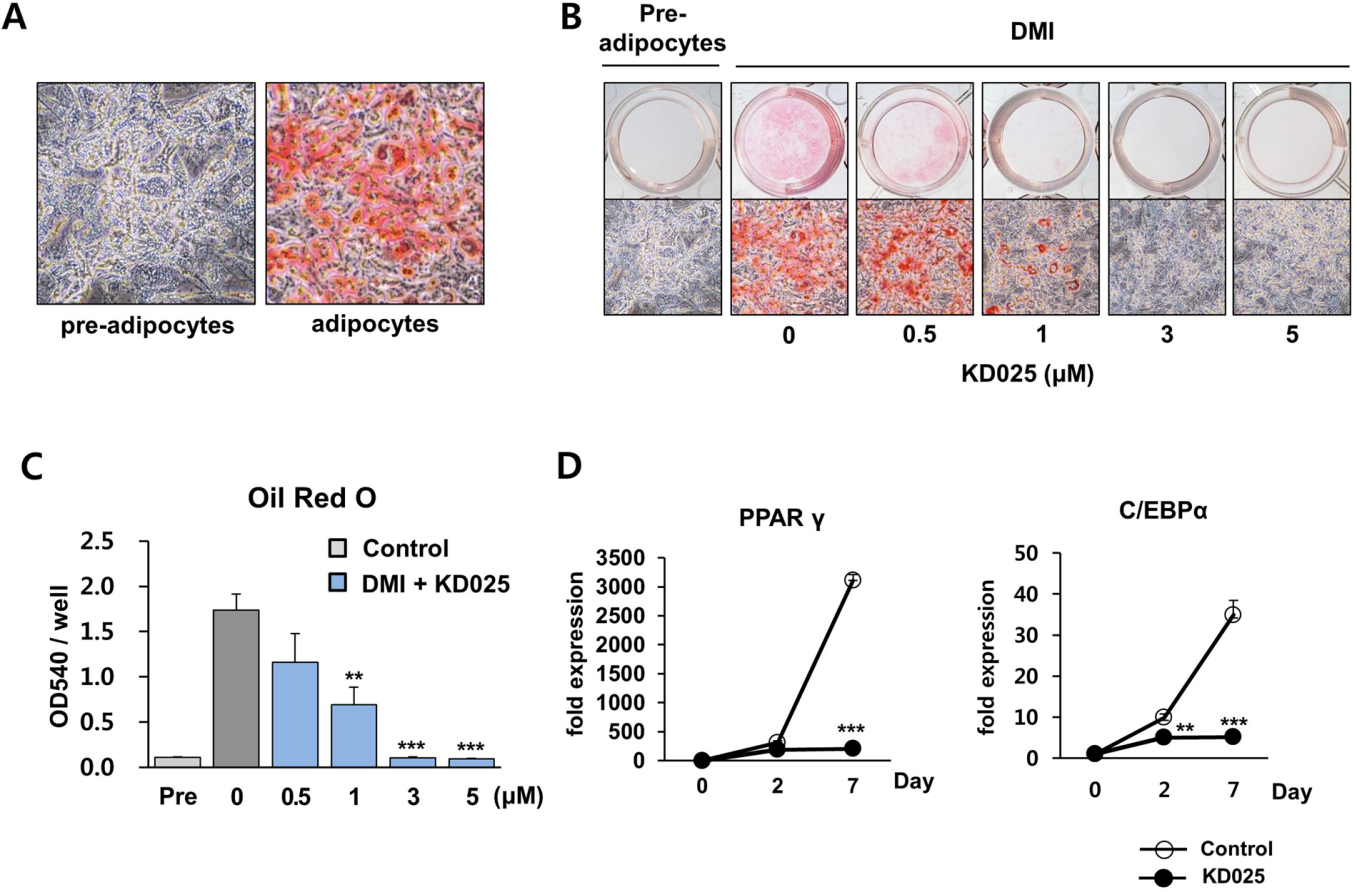
Effect of KD025 on adipogenesis in 3T3-L1 adipocytes. 3T3-L1 cells were differentiated through incubation in DMI (dexamethasone, IBMX, and insulin mixture) with or without KD025. (**A**–**C**) Pre-adipocytes and differentiated adipocytes were stained with Oil Red O at day 8 after the start of differentiation (day 0). (**B**) Concentrations of 0, 0.5, 1, 3, and 5 µM of KD025 with DMI were used to treat cells. Macroscopic and microscopic pictures of cells are shown. (**C**) Lipid accumulation was assessed by measuring absorbance at 540 nm of Oil Red O. **p < 0.01; ***p < 0.001 *vs*. untreated. (**D**) Cells were differentiated with or without 5 µM of KD025 and mRNA expression of *Pparg* and *Cebpa* was measured by real time PCR at days 0, 2, and 7. The data are the representative from more than three independent experiments. Data are expressed as means ± S.E. based on triplicate. **p < 0.01; ***p < 0.001 *vs*. the corresponding control condition.

The Rho-ROCK signaling pathway plays an important role in adipocyte differentiation, and ROCK2 has been suggested as the messenger and transducer of the anti-adipogenic activity of Rho. To explore the ROCK2-specific role in adipogenesis, we suppressed ROCK2 activity during adipocyte differentiation of 3T3-L1 cells using KD025, a ROCK2-specific inhibitor^10^. Oil Red O staining was performed to visualize lipid accumulation in 3T3-L1 adipocytes. Fat accumulation was visualized as a red color at day 8 after treatment with the differentiation cocktail (DMI) consisting of 1 μM dexamethasone, 0.5 mM 3-isobutyl-1-methylxanthine (IBMX), and 5 μg/ml insulin^11^. (day 0) (Fig. 1A). When KD025 was administered with DMI from day 0 to day 7, the amount of fat significantly decreased in a dose-dependent manner (Fig. 1B,C). These data were inconsistent with expectations based on previous studies showing an anti-adipogenic role for the RhoA-ROCK signaling pathway^20–23^. With a relatively low dose of KD025 (3 and 5 μM), lipid storage was undetectable in the cells. To determine if this inhibitor disturbs adipocyte differentiation, mRNA expression of key pro-adipogenic transcription factors PPARγ and C/EBPα was measured by qRT-PCR analysis. As shown in Fig. 1D, DMI medium induced significant increases in the mRNA expression of *Pparg* and *Cebpa* genes. In contrast, KD025 significantly suppressed expression of those key regulators. These findings indicate that KD025 inhibits fat accumulation in 3T3-L1 cells treated with DMI by inhibiting adipocyte differentiation.

### Comparison of the effect of ROCK inhibitors on adipogenesis

**Figure 2:**
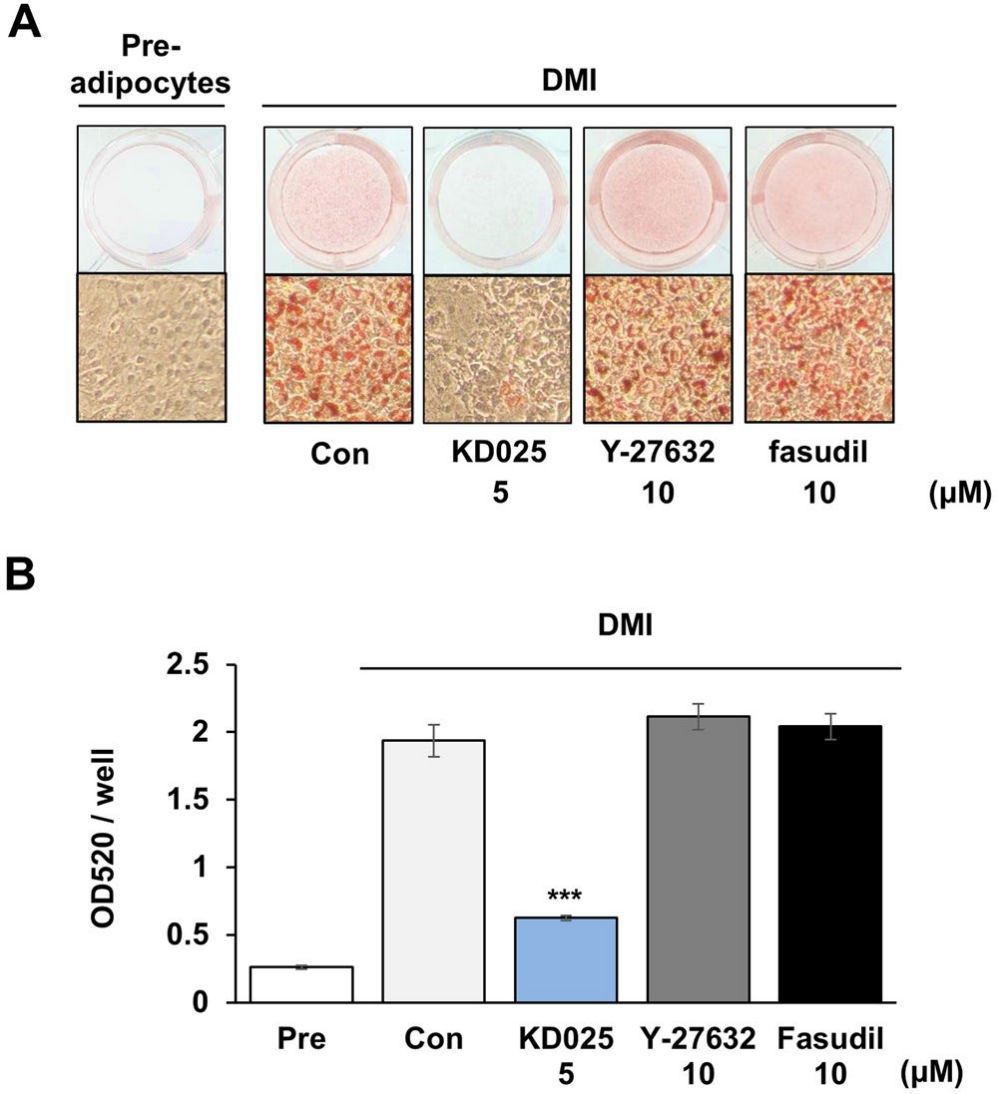
Comparison of the effects of ROCK inhibitors during 3T3-L1 adipogenesis. (**A**) 3T3-L1 cells were differentiated by incubation in DMI with KD025, Y-27632, or fasudil, as indicated, and stained with Oil Red O on day 8. (**B**) Lipid accumulation was assessed by measuring absorbance at 520 nm of Oil Red O. The data are the representative from more than three independent experiments. Data are expressed as means ± S.E. based on triplicate. ***p < 0.001 *vs*. DM control.

A previous study showed that well-known ROCK pan-inhibitors (Y-27632 and fasudil) promote adipogenesis in 3T3-L1 cells when they are administered with dexamethasone- and IBMX-containing media (DM)^19^. We further compared the effects of KD025 in combination with pan-inhibitors on adipogenesis to confirm the known effect of pan-inhibitors and to identify whether the anti-adipogenic activity of KD025 resulted from ROCK inhibition. Inhibitors were treated with differentiation media (DMI) from day 0 to day 8. Compared to the apparent inhibitory effect of KD025 on adipogenesis, Y-27632 and fasudil did not suppress differentiation (Fig. 2A,B). This discordant effect of KD025, when compared to that of pan-inhibitors, suggests that KD025 might inhibit adipogenesis through a ROCK-independent pathway.

### KD025 suppresses expression of late adipogenic and lipogenic genes but not early adipogenic genes

**Figure 3:**
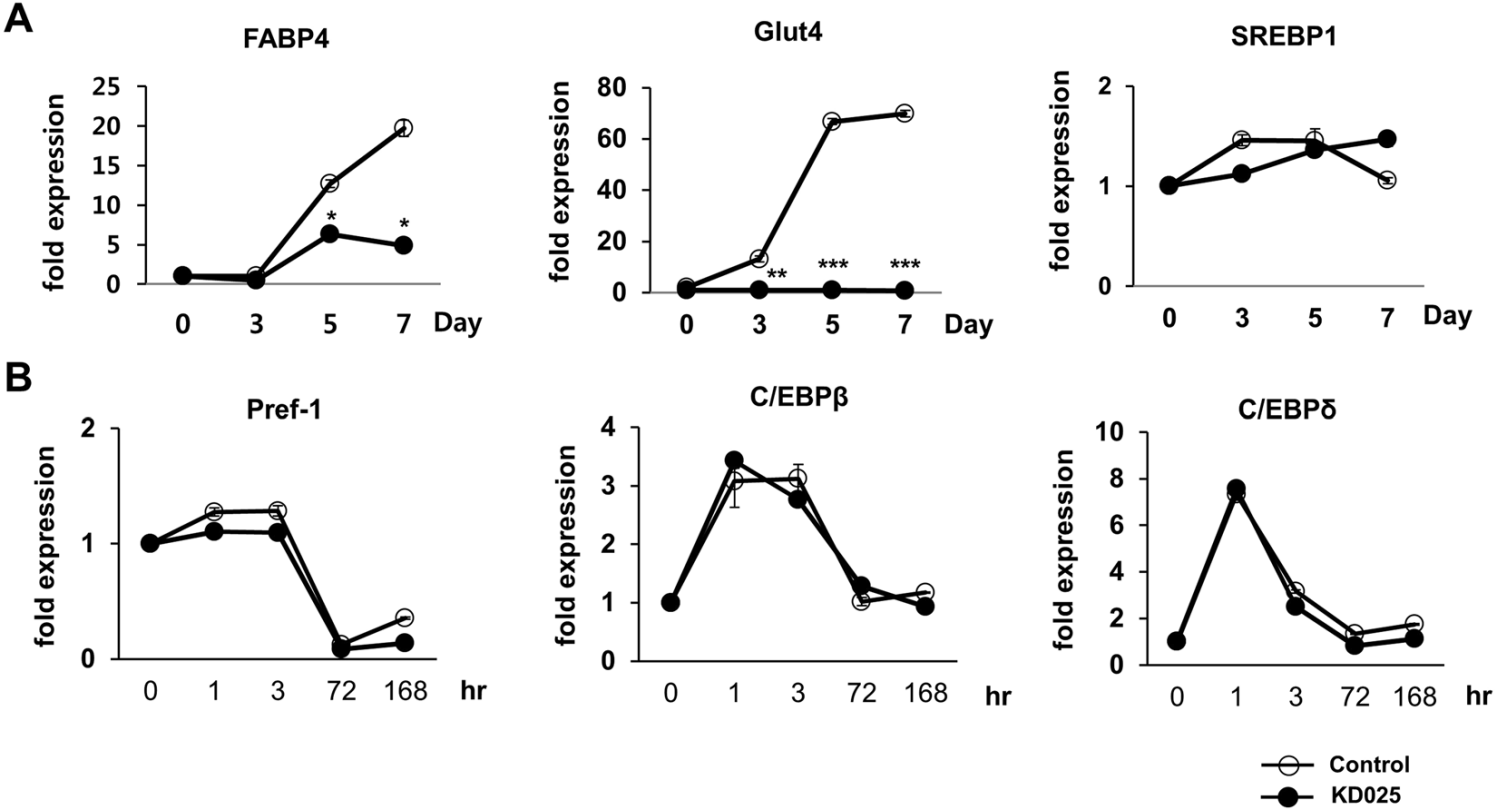
Effects of KD025 on the expression of adipogenic and lipogenic genes. 3T3-L1 cells were differentiated through incubation in DMI with or without KD025 (5 µM) for the indicated time points and mRNA expression was measured by real time PCR. (**A**) Lipogenic genes: *Fabp4, Slc2A4, and Srebp1*. *p < 0.05; **p < 0.01; ***p < 0.001 *vs*. the corresponding control condition. (**B**) *Pref1* and early activated genes: *Cebpb* and *Cebpd*. The data are the representative from more than three independent experiments. Data are expressed as means ± S.E. based on triplicate.

Adipocyte differentiation requires a series of critical gene expression events^24–27^. This process starts with the induction of C/EBPβ and C/EBPδ at an early stage, during which cells begin to express the key adipogenic transcription factors PPARγ and C/EBPα. Once expressed, C/EBPα activity initiates a positive feedback loop affecting PPARγ activity and these two factors enhance the expression of the other to maintain a differentiated state^28,29^. Because our results showed that KD025-exposed cells can significantly block *Pparg* and *Cebpa* expression, we examined the effects of KD025 on the expression of lipogenic transcription factors by qRT-PCR. KD025 treatment significantly suppressed *Fabp4* and *Slc2A4* expression, when compared to expression in the control, whereas irregular patterns were observed for *Srebf1* (Fig. 3A). However, the expression of *Dlk1*, which encodes pre-adipocyte factor 1 (Pref-1), in addition to that of two early activated adipogenic genes, *Cebpb* and *Cebpd*, was not affected by KD025 treatment (Fig. 3B). Pref-1 is an excellent marker of pre-adipocytes, and inhibition of adipogenesis by Pref-1 has been well established *in vitro* as well as *in vivo*^30,31^. These results suggest that KD025 might not regulate its targets during the early stage, but could suppress its targets during the intermediate or late stage of adipogenesis.

### KD025 inhibits adipogenic events in 3T3-L1 cells during the intermediate stage

**Figure 4:**
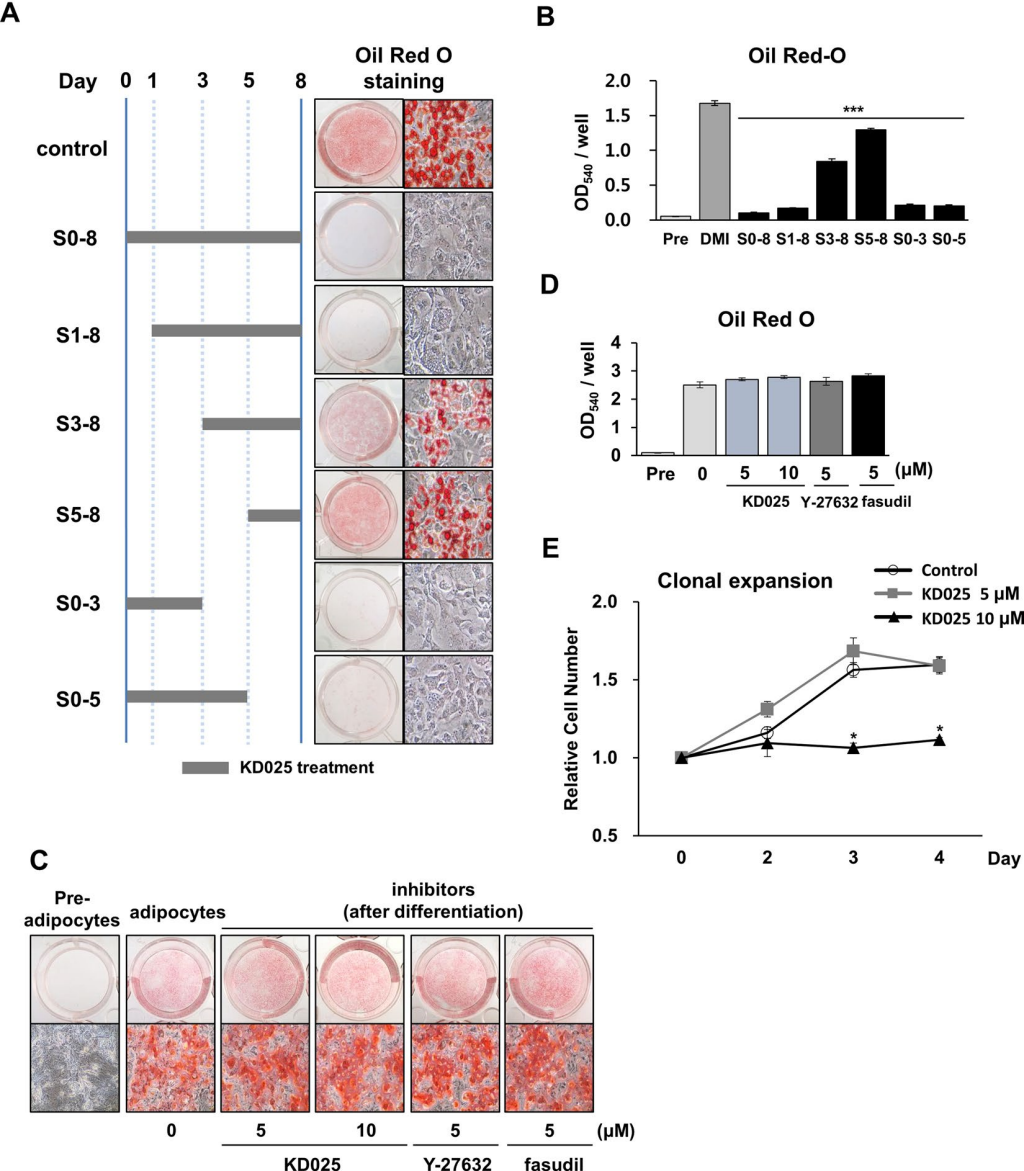
Phase-specific effect of KD025 on adipogenesis. (**A**,**B**) 3T3-L1 cells were differentiated through incubation in DMI with 5 µM KD025 at the indicated time points and stained with Oil Red O at day 8. S0-8; KD025 was added to the growth media from day 0 to day 8, S1-8, from day 1 to day 8, S3-8, from day 3 to day 8, S5-8, from day 5 to day 8, S0-5, from day 0 to day 5. The differentiated adipocytes were stained with Oil Red O on day 8, and microscopic pictures of cells were taken. (**B**) Lipid accumulation was assessed. ***p < 0.001 *vs*. untreated. (**C**,**D**) Differentiated 3T3-L1 adipocytes (day 8) were treated with ROCK inhibitors at the concentrations indicated for 2 days, and lipid accumulation was measured. (**E**) Cells were differentiated in DMI medium with KD025 for 0, 2, 3 and 4 days, trypsinized, and counted. *p < 0.05 *vs*. untreated. The data are the representative from more than three independent experiments. Data are expressed as means ± S.E. based on triplicate.

Our work showed that KD025 significantly decreases the expression of early activated genes (Fig. 3). To determine the mechanism of such inhibitory effects, cells were exposed to KD025 at various time points after the initiation of differentiation (Fig. 4A). As shown in Fig. 4A,B, lipid content was efficiently decreased after exposure to KD025 during the early-to-intermediate stages (days 0–3), whereas a lesser effect emerged during the late stages (days 3–8 and days 5–8). Differentiation was effectively inhibited by exposure to KD025 at a very early stage even without continued treatment. These data indicate that KD025 mainly targets the intermediate stage (days 1–3) of adipogenesis, which is consistent with KD025’s temporal effect on pro-adipogenic genes (Fig. 3). To determine whether KD025 affects lipid storage after differentiation, we examined the effect of KD025 on post-adipocytes. As shown in Fig. 4C,D, no change emerged in lipid content when differentiated cells were exposed to KD025. We further examined the effect of KD025 on mitotic clonal expansion, which is an early event during 3T3-L1 cell adipogenesis. KD025 at 5 and 10 µM was added to the DMI differentiation medium, and cells were counted. Cells exposed to 5 µM of KD025 on the second, third, and fourth days did not show any significant changes in mitotic clonal expansion. In contrast, 10 µM of KD025 resulted in no increase in the number of cells, thereby indicating an absence of mitotic clonal expansion. Because KD025 inhibited adipogenesis in 3T3-L1 cells at a concentration of less than 5 µM, the inhibitory effect on cell growth at 10 µM might have resulted from cytotoxicity, unrelated to its anti-adipogenic role (Fig. 4E).

### KD025 negatively regulates adipogenesis without suppression of the insulin signaling pathway

**Figure 5:**
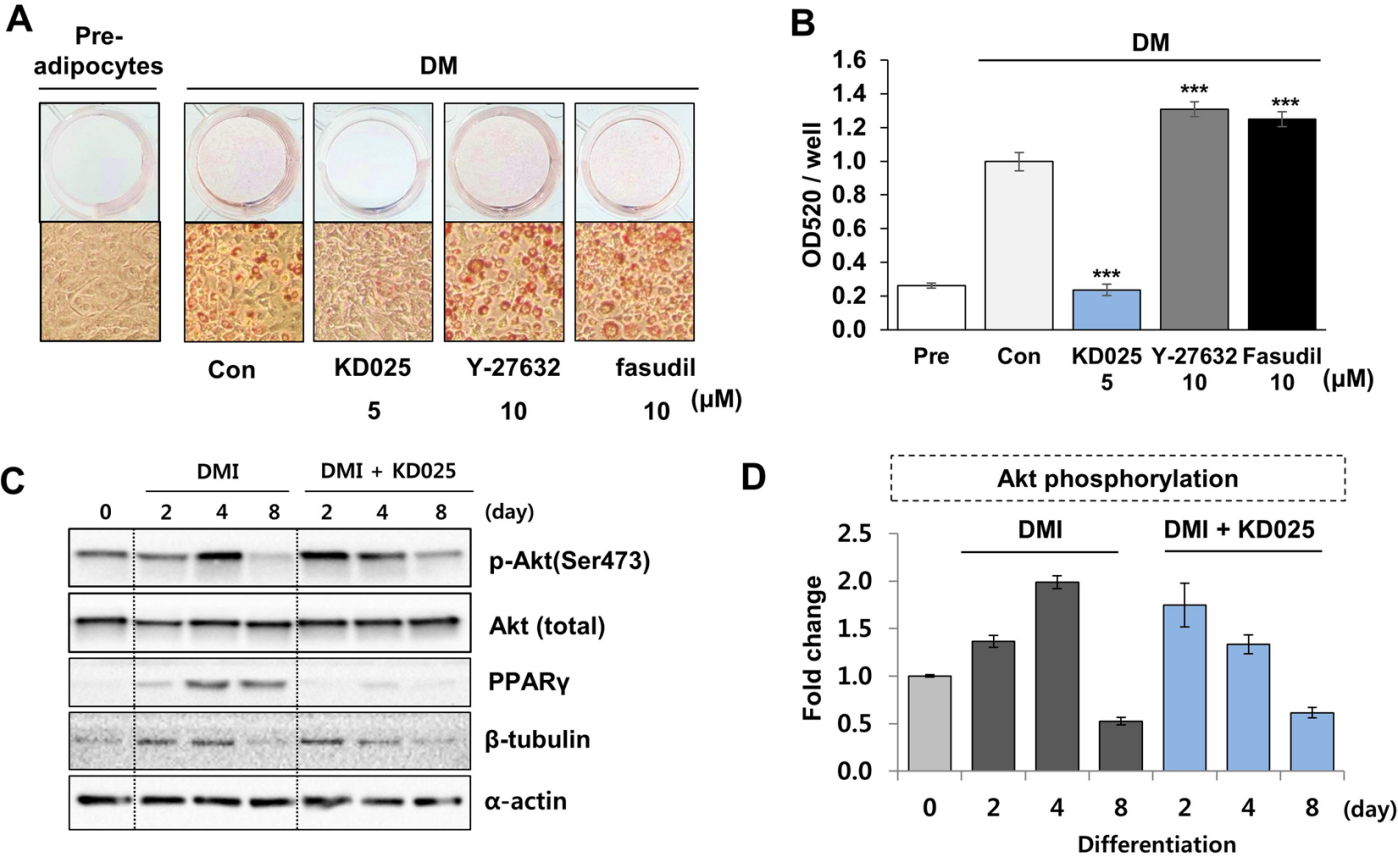
Effects of ROCK inhibitors on insulin action and signaling. (**A**) 3T3-L1 cells were differentiated through incubation in DM (without insulin) with KD025, Y-27632, or fasudil, as previously indicated, and stained with Oil Red O on day 8. (**B**) Lipid accumulation was assessed by measuring the absorbance at 520 nm of Oil Red O. ***p < 0.001 *vs*. DM control. (**C**,**D**) 3T3-L1 cells were differentiated in DMI-containing media with or without KD025 for 2, 4, or 8 days. (**C**) Cells lysates were subjected to SDS-PAGE and immunoblotted with anti-phospho-Akt (Ser473) and total Akt antibodies. PPARγ was used as a differentiation marker, and β-tubulin and α-actin as loading controls. (**D**) The level of phosphorylated Akt at Ser473 residue was illustrated during adipogenesis. *p < 0.05; **p < 0.01; ***p < 0.001 *vs*. insulin + vehicle untreated. The data are the representative from more than three independent experiments. Data are expressed as means ± S.E. based on triplicate.

Insulin is a key inducer of lipogenesis and adipocyte differentiation^32^. As noted above, Y-27632 or fasudil showed an insulin-like differentiation-promoting effect in 3T3-L1 cells^19^. Y-27632 inhibited insulin-induced Ser^632/635^ phosphorylation of IRS-1 and enhanced insulin-stimulated Akt phosphorylation in 3T3-L1 pre-adipocytes^19^. To evaluate the effects of KD025 on insulin signaling, we incubated DM medium with or without ROCK inhibitors and measured lipid accumulation. In our study, in the absence of insulin, KD205 reduced lipid content significantly (Fig. 5A) as well as under insulin stimulation. In contrast, Y-27632 and fasudil enhanced lipid accumulation by around 30% and 25% respectively (Fig. 5A,B), although the effect was not as strong as the previous report^19^. Because serum is a key factor affecting growth-related signal, we considered that the type of serum would contribute to the occurrence of this difference between studies. To test this inference, we analyzed differentiation with calf serum (CS; same as the previous study^19^) instead of newborn calf serum (NBCS). Regardless of the kinds of serum, three ROCK inhibitors showed similar efficacies upon cell differentiation (Supplemental Figures S1A and S1B).

To identify how KD025 regulates the insulin signaling pathway, we analyzed the effect of KD025 on the pathway by immunoblot analysis. During adipogenesis, Akt activity was upregulated at day 2 and 4 of adipogenesis then downregulated at day 8 (Fig. 5C,D). Data show that KD025 treatment modulated Akt activity during the early-to-intermediate stage however, the effect was too irregular to derive any obvious pattern.

To confirm that the Akt-inhibitory effect of KD025 depends on normally-fed cells, we treated three different cell lines with this compound for 1 day in normal conditions (10% FBS) without any starvation/insulin stimulation intervention and examined Akt phosphorylation by immunoblotting. Normally cultured cells (3T3-L1, L6, HeLa) under serum activation showed no change in Akt phosphorylation by KD025 in which Akt might be highly activated (Supplemental Figure S2).

### Regulation of ROCKs during adipogenesis of 3T3-L1 cells

**Figure 6:**
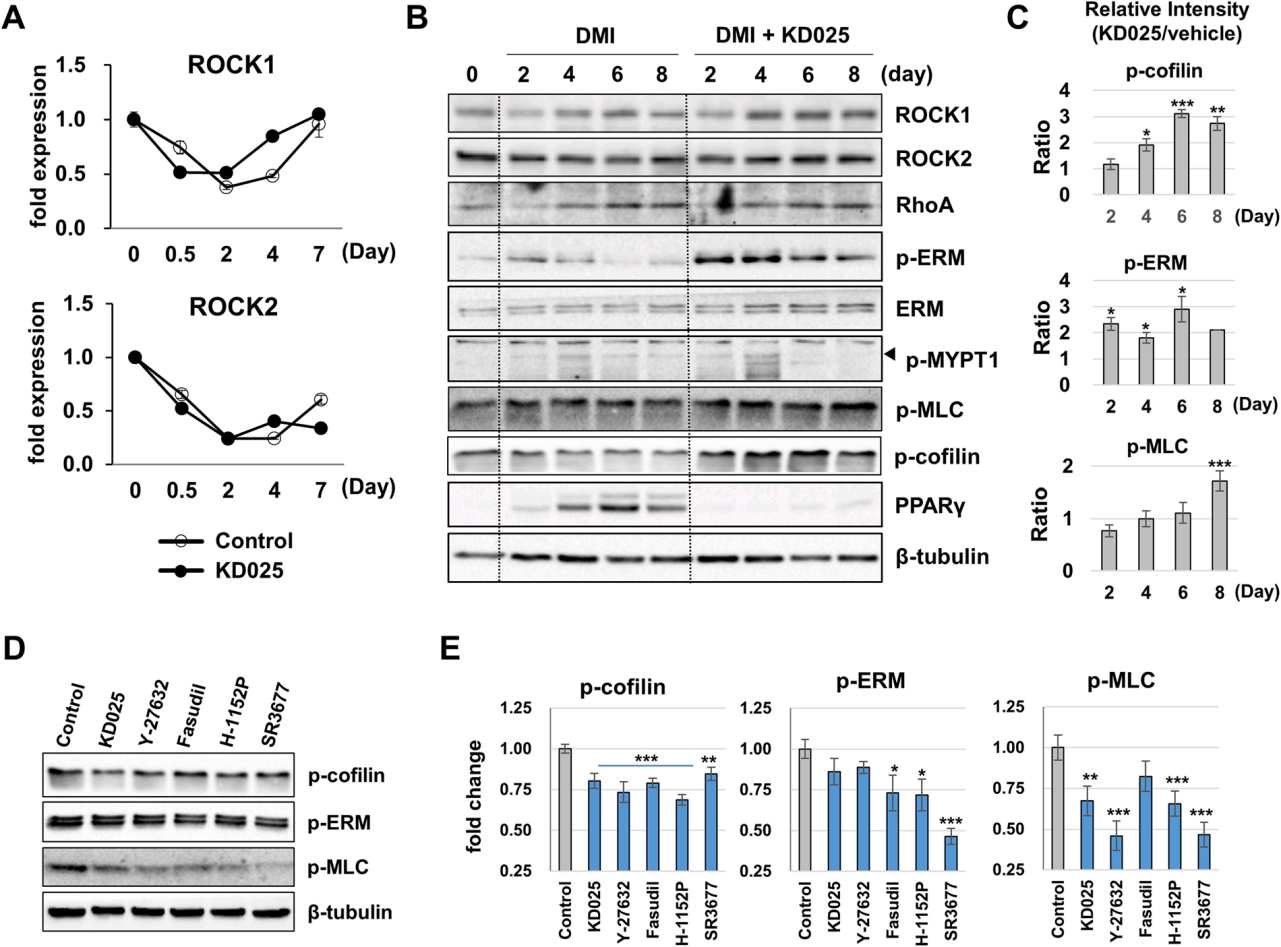
Regulation of ROCKs during adipogenesis. (**A**–**C**) 3T3-L1 cells were differentiated through incubation in DMI with or without KD025 for the indicated time points. (**A**) mRNA expression levels of ROCK1 and ROCK2 were measured by qRT-PCR. (**B**) Protein expression levels of ROCK1, ROCK2, p-cofilin, p-MLC, p-MYPT1, p-ERM, ERM and RhoA were analyzed by immunoblot. PPARγ was used as a marker of adipogenesis and β-tubulin as a loading control. (**C**) The relative intensity of phosphorylation levels of cofilin, ERM, and MLC were compared between vehicle- and KD025-treated conditions. *p < 0.05; **p < 0.01; ***p < 0.001 *vs*. the corresponding control. (**D**,**E**) 3T3-L1 pre-adipocytes were treated with KD025 (7 µM), Y-27632 (10 µM), fasudil (10 µM), H-1152P (3 µM), or SR3677 (5 µM) for one day. The phosphorylation levels of cofilin, ERM, and MLC were measured by immunoblot. β-tubulin was used as a loading control. *p < 0.05; **p < 0.01; ***p < 0.001 *vs*. vehicle-treated. The data are the representative from more than three independent experiments. Data are expressed as means ± S.E. based on triplicate.

Numerous studies consistently show that ROCKs might be negative regulators in adipogenesis. To explain further the mechanism of the anti-adipogenic role of KD025, we tested whether downregulation of ROCKs’ activity is required for adipogenesis and regulation of ROCKs’ activity mediates the anti-adipogenic effect of KD025. To analyze transcriptional level of ROCKs during adipogenesis of 3T3-L1 cells, we measured mRNA levels of ROCKs during adipogenesis with or without KD025 using qRT-PCR. On both isotypes, the mRNA expression was lowest at day 2 and gradually recovered during which KD025 did not significantly affect the pattern (Fig. 6A). We further confirmed this by obtaining gene-expression data from the NCBI Gene Expression Omnibus (GEO) database (GSE id: 6795). Our analysis showed that *Rock1* and *Rock2* mRNA expressions were sustained in slightly lower levels during differentiation (Supplemental Figure S3). The expression level change was moderate through the period compared to level change of some noticeable genes, such as *Pparg*. When protein levels were measured by immunoblotting, ROCK1 decreased at day 2 and recovered at later stages whereas ROCK2 was maintained in slightly lower quantities during adipogenesis (Fig. 6B). To evaluate the role of ROCKs we further explored the activities of downstream (cofilin, ERM, MLC, and MYPT1) and upstream (RhoA) molecules in RhoA-ROCK axis. By DMI stimulation, cofilin, ERM and MYPT1 phosphorylations were upregulated at an early stage and then gradually downregulated; this pattern was not coincident with the pattern of ROCKs levels (Fig. 6B). In particular, phosphorylations of cofilin and MLC were not suppressed but rather enhanced by KD025 treatment during adipogenesis (Fig. 6C) while they were suppressed significantly in 3T3-L1 pre-adipocytes. These findings suggest the possibility that these proteins might be stimulated by other regulators rather than ROCKs.

To see if this activation by KD025 is an adipogenesis-specific event, we further tested the effect of KD025 on cofilin, ERM and MLC proteins in normally fed cells. KD025 decreased the phosphorylation of cofilin (0.80-fold, p < 0.005) and MLC (0.67-fold, p < 0.01) significantly indicating that KD025 ROCK’s pathway in a normal way in a generally cultured condition (Fig. 6D,E). Furthermore, other ROCK inhibitors also had a similar effect on the phosphorylation levels with slightly differential spectrums. These findings indicate that KD025 may affect RhoA-ROCK signaling in different ways according to the environmental context of cells.

### KD025 regulates adipogenesis partially in a ROCK-independent way

**Figure 7:**
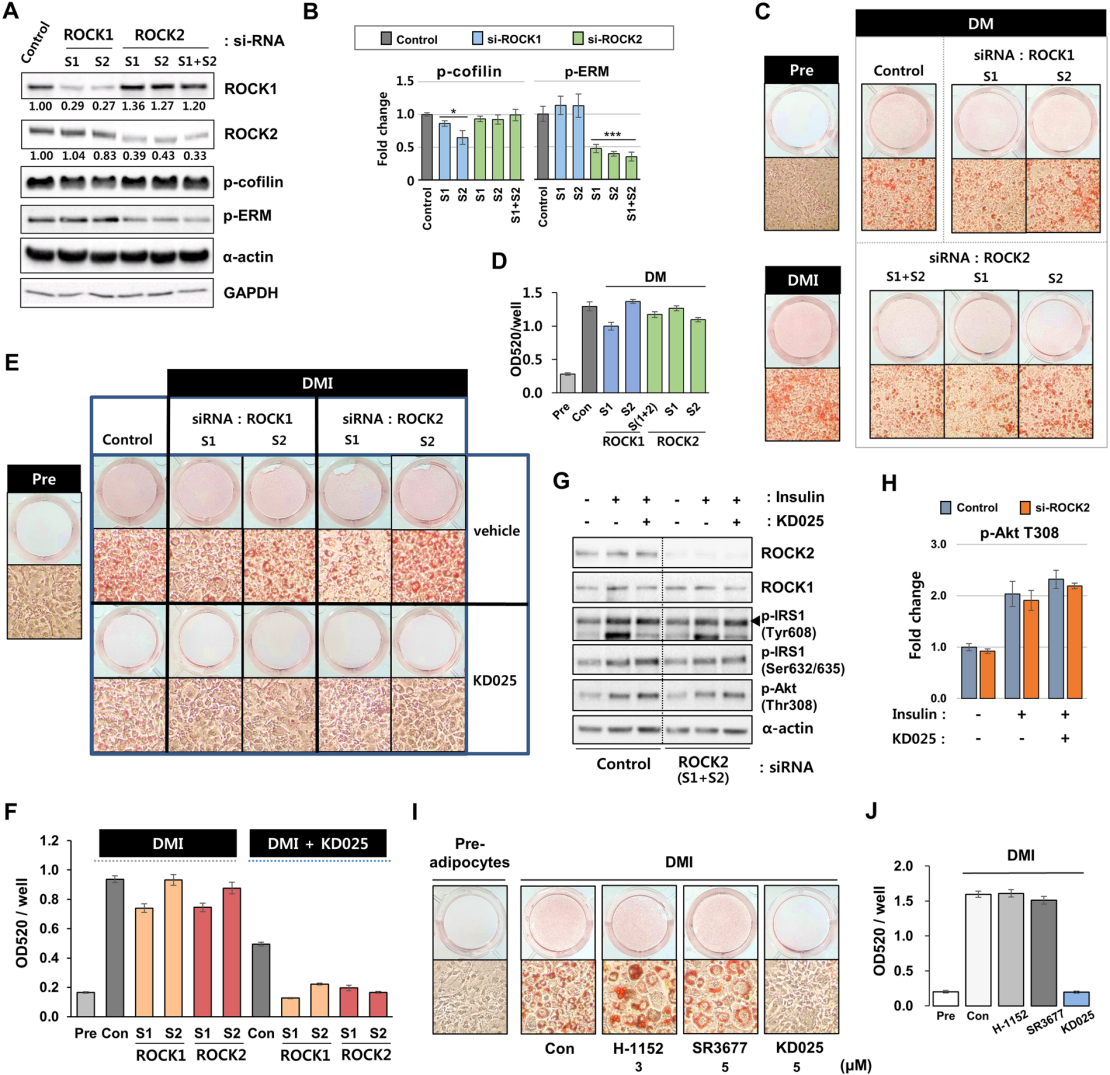
ROCK-independent action of KD025 on adipogenesis. (**A**,**B**) 3T3-L1 cells transfected with two different sequences (S1 and S2) of ROCK1 or ROCK2 siRNA and incubated for 2 days to reach confluence. (**A**) Cell lysates were subjected to SDS-PAGE and analyzed using anti-ROCK1 and anti-ROCK2 antibodies to see the depletion of each isoform. The phosphorylation levels of cofilin and ERM were measured using phospho-specific antibodies. GAPDH and α-actin were used as loading controls. (**B**) The phosphorylation level change of cofilin and ERM by gene knockdown of ROCK 1 and 2 was depicted as fold changes. *p < 0.05; **p < 0.01; ***p < 0.001 *vs*. vehicle-transfected. (**C** and **D**) Transfected cells were grown to confluence and maintained for 2 days and then differentiated through incubation with DM-containing medium for 8 days. (**C**) Cells were stained with Oil Red O. Macroscopic and microscopic pictures of cells are shown. (**D**) Lipid accumulation was assessed by measuring absorbance at 520 nm of Oil Red O. (**E**–**G**) Transfected cells with ROCK siRNAs were differentiated through incubation with DMI for 8 days with or without KD025 (5 µM). (**E**) Cells were stained with Oil Red O. Macroscopic and microscopic pictures of cells are shown. (**F**) Lipid accumulation was assessed by measuring absorbance of Oil Red O. (**G**,**H**) Insulin signaling pathway was inspected with lysates from ROCK2 knocked-down pre-adipocytes cells (S1 + S2) with or without KD025 treatment. Immunoblot analysis was undergone with antibodies for phospho-IRS-1 (Tyr608 and Ser632/635), phospho-Akt (Thr308), Akt, ROCK1, and ROCK2. α-actin was used as a loading control. The relative level of p-Akt (Thr308) was assessed as fold changes compared to insulin-/KD025-untreated control cells. (**I**,**J**) The effect of H-1152 and SR3677, potent ROCK inhibitors, was analyzed on 3T3-L1 differentiation. (**G**) Cells were stained with Oil Red O and microscopic images are displayed. (**H**) Lipid accumulation was assessed of Oil Red O. The data are the representative from more than three independent experiments. Data are expressed as means ± S.E. based on triplicate.

To test the role of ROCKs in 3T3-L1 differentiation, we knocked down ROCK1 and ROCK2 using siRNA technology and measured the effect on lipid accumulation. In this study, we knocked down two different sequences for each ROCK isotype to minimize misinterpretation resulting from non-specific off-target effects (Fig. 7A). The phosphorylation of cofilin and ERM was downregulated by knockdown of ROCK1 and ROCK2 isoform respectively (Fig. 7A,B). When ROCKs-knockdowned 3T3-L1 cells were incubated in DM media, fat accumulation was only slightly changed according to sequences, considered as a non-specific effect of siRNA (Fig. 7C,D). However, we could not observe any increase in adipogenesis. When these cells were stimulated with DMI, they also showed slight changes in differentiation levels according to types of sequences (Fig. 7E,F). Of note, regardless of isotype of ROCK depleted, KD025 efficiently suppressed DMI-stimulated differentiation. To see the effect of KD025 on insulin signaling, ROCK2-depleted preadipocytes in confluence were pre-incubated with KD025, then starved and stimulated with insulin. Immunoblot analysis showed that ROCK2 depletion had little effect on phosphorylation of Akt (Thr308) and IRS1 (Tyr608 and Ser632/635). KD025 treatment in ROCK2-depleted cells showed slightly increased Akt phosphorylations (Fig. 7G,H) but not significant in both control and ROCK2-depleted cells (p = 0.18 and 0.07 respectively). These results indicate KD025 might inhibit differentiation irrelevant to the activity of ROCK2.

Because our results indicate ROCK activity is not critical for 3T3-L1 adipogenesis, we further evaluated the role of ROCKs using two potent ROCK inhibitors, H-1152P and SR3677; H-1152P is highly potent for ROCK2 (Ki_50_ (ROCK2) = 1.6 nM)^33^ and SR3677 is moderately specific on ROCK2 (IC_50_ (ROCK1) = 56 nM, IC_50_ (ROCK2) = 3.2 nM)^34^. 3T3-L1 cells treated with these inhibitors were differentiated without any noticeable change in total fat accumulation (Fig. 7I,J). Uniquely, the cells treated with these inhibitors were much larger in cell size and had more lipid droplets per cell.

Collectively, these results raise questions about whether ROCKs play key roles in adipogenesis, or whether their inhibitions might be compensated by other factors. Of note, our data strongly suggest KD025 may reveal an anti-adipogenic effect at least, partially independent of the modulation of ROCK activity.

### KD025 does not inhibit actin cytoskeleton formation during adipocyte differentiation

**Figure 8:**
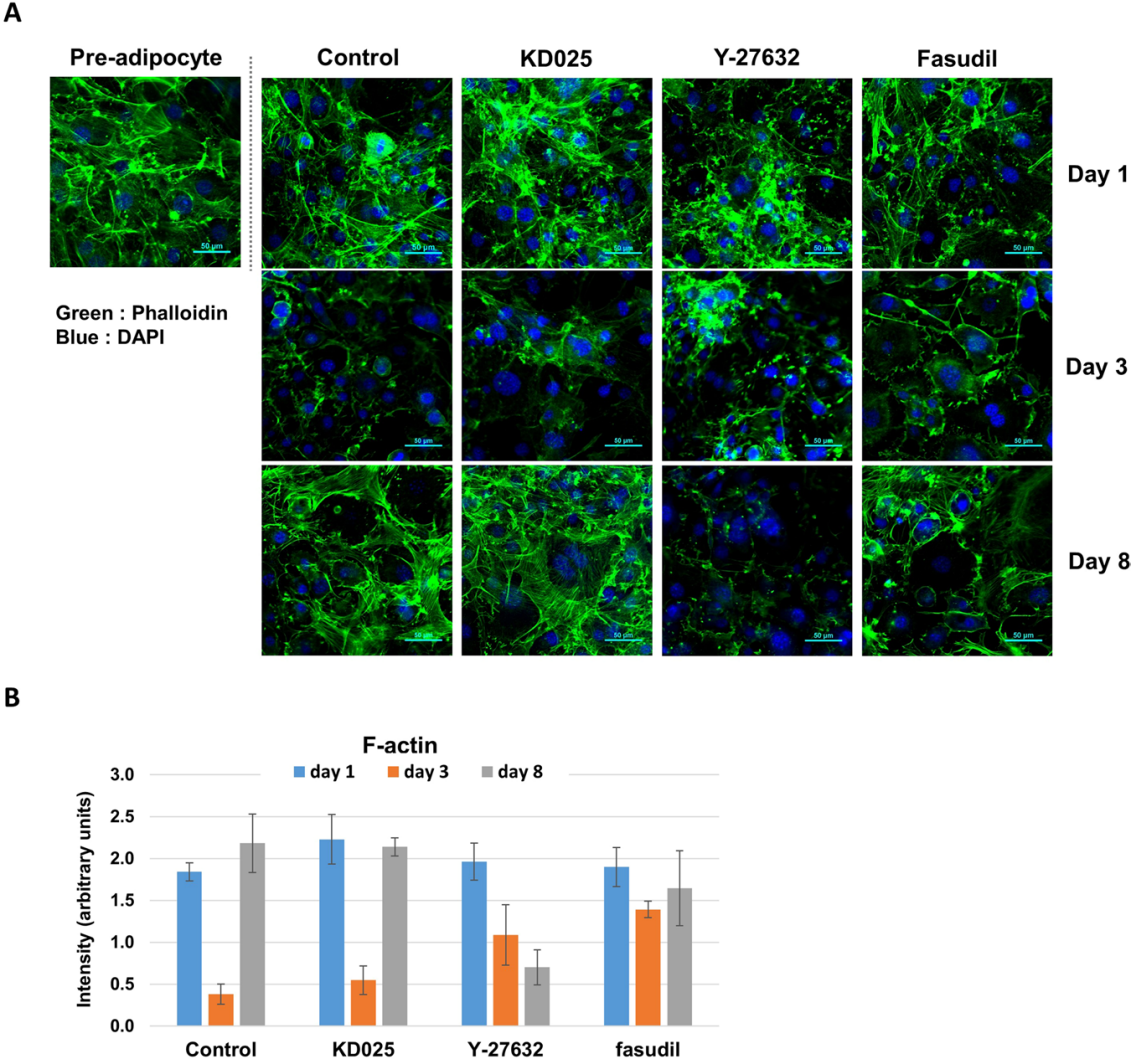
Effects of KD025 on actin cytoskeleton during adipogenesis. (**A**) 3T3-L1 cells were differentiated through incubation with DMI in the presence of 10 µM of KD025, Y-27632, or fasudil. Cells were fixed on day 1, 3, or 8 after the start of differentiation, and were probed for F-actin (green) using phalloidin and the nucleus (blue) with DAPI. Pre-adipocyte was stained for comparison (left). The horizontal bar represents 50 μm. (**B**) The level of total F-actin was measured and represented in arbitrary units. The average intensity level per cell was derived by obtaining the sum of F-actin intensity from multiple independent images and then by dividing the sum with total cell number (n ≥ 80). Data are expressed as means ± S.E.

A rounded morphology and loss of actinomyosin fibers are critical for adipogenesis^21,35,36^. These characters are associated with Rho-ROCK signaling through multiple mechanisms, and the effects of Y-27632 on actin fiber formation in various cell types are well documented^11,37–39^. To identify the effect of KD025 on actin fiber formation during adipogenesis, we treated post-confluent 3T3-L1 cells at different differentiation stages with KD025. After administration with the DMI differentiation cocktail, actin stress fibers (green) were suppressed during the intermediate-late stage (control, day 3). In the late stage (day 8), cortical actin developed in differentiated cells but stress fibers were recovered from the remaining undifferentiated cells (Fig. 8A). At day 3, most cells treated with KD025 lost actin stress fibers as untreated cells did, but actin stress fibers were recovered from those cells on day 8. To quantify the level of actin fibers in cells, we measured the intensity of total F-actin per cell. The results show that KD025-treated cells recovered the total F-actin to the comparable level to pre-adipocytes (Fig. 8B). In contrast, Y-27632 and fasudil treatment resulted in a significant loss of actin fiber structures (Fig. 8A,B) on day 8. Meanwhile, KD025 did not change the total level of F-actin structures in the early-to-intermediate stage, during adipogenesis. These findings indicate that KD025 does not inhibit or accelerate actin stress fiber formation of which the loss is required for the progression of adipogenesis; thus, the anti-adipogenic effect of KD025 can be maximized during adipogenesis.

## Discussion

ROCKs are found to inhibit adipogenesis by multiple means, although only a few studies have provided direct evidence of this. Nevertheless, the anti-adipogenic roles of ROCK are generally accepted, along with supportive evidence that Rho proteins and p190Rho-GAP, both of which closely related to ROCK function, negatively regulate adipogenesis. Previous studies showed that the Rho-ROCK pathway inhibits adipogenic determination^2^. In MSCs, cellular confluency of spindle fibroblasts induces rounded cell morphology through the inactivation of Rho-ROCK activity and the loss of actinomyosin fiber formation, required for adipogenesis^21,36^. Furthermore, several studies using ectopic expression of constitutively active Rho, p190B RhoGAP-deficient mice, and pan-inhibitors (Y-27632 and fasudil) showed insulin-like and pro-adipogenic effects of the ROCK signaling pathway^18,19,21^.

Currently, several mechanisms underlying the effects of Rho-GTPase and ROCKs on anti-adipogenic action have been suggested. First, ROCKs deliver Rho-mediated function by inhibiting the expression of pro-adipogenic WNT genes while elevating anti-adipogenic WNT genes. Second, ROCKs are key regulators of actinomyosin formation which is a key determinant of adipogenesis. Third, ROCK inhibits the action of insulin required for adipogenesis. Fourth, ROCK2 is a relevant messenger of Rho signaling for the inhibition of adipogenesis^19^. However, our current knowledge is still incomplete regarding the integration of these data, and more studies using diverse approaches are required; adipogenesis studies using ROCK1- or ROCK2-deficient mice might provide comprehensive knowledge in understanding isoform-specific roles in adipogenesis. In this regard, our previous study demonstrated that ROCK1 is a negative regulator in insulin signaling using adipose tissue specific ROCK1-lacking mice^40^. However, this study had a limitation in that ROCK1’s role was not intensely addressed on adipogenesis; ROCK1 might be normally expressed during adipogenesis and be deleted after adipocyte maturation by adiponectin-Cre recombinase. In the future, it would be highly desirable to study using adipose tissue precursor cell-specific ROCK deleted animals if available.

Previously, most studies suggested that ROCKs have common characteristics as mediators of RhoA, but these studies have not considered their distinctive roles. However, as predicted from the different protein sequences, recent evidence has increasingly suggested that the two isoforms differ in terms of binding partners, spectra of cellular function, and regulatory mechanisms. ROCK-deficient mice have their own isoform-specific phenotypes, meaning they have isoform-specific roles *in vivo*^41^. These two isoforms tend to exhibit isoform-specific effects by interacting with distinct partners^11,42–46^. In this respect, the use of ROCK2-specific inhibitors can be a promising approach, as presented in a study performed by Zandi *et al*. to identify ROCK isoform-specific roles in the polarization of macrophages associated with age-related macular degeneration (AMD)^10^. In another study, ROCK2 was suggested as a exacerbating factor in adipose tissue to cause obesity-induced insulin resistance as evidenced by the fact that adipocyte hypertrophy and inflammatory cytokines might mediate this exacerbation^47^.

Although ROCK2 is a suggested regulator of adipogenesis, no studies have been conducted using a ROCK2-specific inhibitor to examine the isoform-specific role of ROCK during adipogenesis. KD025 is a ROCK2-specific inhibitor that has received much attention recently^10^. Y-27632 and fasudil have been mainly documented as inhibitory agents of muscle contraction; in most studies, the discrete roles of ROCK isoforms were not studied. However, recently, isoform-specific inhibitors have been receiving greater attention as isoform-specific physiological roles of ROCK are discovered^10,11,40,47–50^. Among isoform-specific inhibitors of ROCK, KD025 exhibits ROCK2-specific inhibitory activity. The median inhibitory concentration (IC_50_) for ROCK2 is 105 nM, whereas it is 24 μM for ROCK1 in a cell-free system^51^.

**Figure 9:**
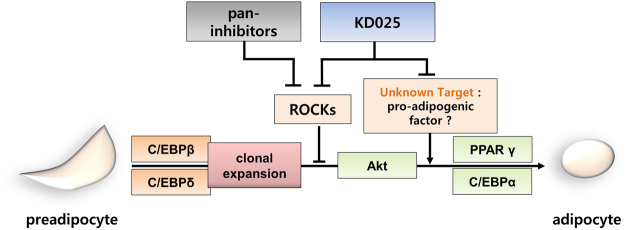
Model of the effects of KD025 on 3T3-L1 adipocyte differentiation. KD025 inhibits adipocyte differentiation in 3T3-L1 cells, regulating the intermediate stage of this process. Differentiation is not promoted by the suppression of ROCK2 activity via KD025. This anti-adipogenic effect of KD025 might be mediated by the suppression of an unknown key pro-adipogenic regulator.

Based on previous studies, we expected KD025, a ROCK2-specific inhibitor, to promote adipogenesis in 3T3-L1 cells. However, this was not the case. Rather, we found that KD025 actually has anti-adipogenic effects. This finding indicates that a specific indispensable regulator of adipogenesis might be targeted by KD025; thus, the pro-adipogenic effects resulting from ROCK2 inhibition were extinguished. We illustrate this working hypothesis in Fig. 9. Our study shows that key genes such as PPARγ and C/EBPα were significantly blocked by KD025, whereas the expression of early adipogenic genes did not change. Furthermore, terminally differentiated adipocytes were unaffected by KD025 in lipid accumulation, and mitotic clonal expansion did not change. These findings indicate that KD025 regulates its targets during the intermediate stage of adipogenesis. In contrast to our KD025 results, two known ROCK kinase inhibitors (Y-27632 and fasudil) were reported to enhance adipogenesis^19^. Given that KD025 inhibited adipogenesis at the concentration at which ROCK2 activity can be blocked, it is conceivable that KD025 affects adipogenesis by targeting unknown key regulators in a ROCK2-independent manner. This was further confirmed from the finding that ROCK-depletion did not perturb the anti-adipogenic action of KD025 (Fig. 7).

It has been reported that the insulin signaling pathway is important for adipogenesis^52^. Y-27632 upregulated insulin-stimulated IRS-1 and Akt activation in pre-adipocytes^19^. In our study, KD025 elevated IRS-1 phosphorylation in response to insulin. In addition, we noticed that KD025 increased the phosphorylation of Akt during insulin stimulation. This character suggests that anti-adipogenic activity of KD025 may be involved with key regulators that overwhelm the effect of Akt activation. Treatment with pan-inhibitors of ROCK resulted in significant loss of actin fiber structures^11,38,39^. In contrast, KD025-treated cells showed no decrease in stress fiber formation compared to control cells. This suggests KD025 might have minimal effects on adipogenesis of actin stress fiber formation, which should be down-regulated during adipogenesis.

Limitations of the current investigation include the followings: We could not find any concrete role of ROCKs during adipogenesis; the effect of ROCK-knocking down was not as same as the previous report by other group^19^. The target of KD025, which would be an off-target in the original concept, should be clarified to resolve the underlying mechanism. This appears to be an important step in rediscovering novel uses of this chemical.

In conclusion, our findings demonstrate that KD025 inhibits adipocyte differentiation by suppressing insulin-mediated events and the expression of pro-adipogenic genes. These findings identify KD025 as a potentially effective anti-adipogenic agent that could be used to treat obesity in the future. In future studies, it would be interesting to identify the direct target of KD025 and to determine whether this inhibitor protects against the development of obesity *in vivo*.

## Materials and Methods

### Cell culture

The pre-adipocyte 3T3-L1 cell culture line, derived from mouse embryos, was cultured in Dulbecco’s modified Eagle’s medium (DMEM) (Invitrogen, Carlsbad, CA), supplemented with 10% heat-inactivated newborn calf serum (NBCS) (Invitrogen), 100 units/mL of penicillin, and 100 µg/mL of streptomycin (Cellgro, Manassas, VA), in a humidified incubator at 37 °C and 5% CO_2_. Cells were differentiated as described above by adding a differentiation cocktail (DMI).

### ROCK inhibitors

KD025 and fasudil were kindly provided by Dr. Ali Hafez-Moghadam (Brigham & Women’s Hospital, Boston, USA) and purchased from MedChem Express (NJ, USA). Y-27632 was purchased from Selleck Chemicals (Houston, Texas) and H-1152P and SR3677 were from R&D Systems (Minneapolis, Minnesota).

### Oil Red O staining

Fat accumulation in 3T3-L1 cells was assessed with the fat-soluble dye Oil Red O (Sigma-Aldrich, St Louis, USA). Differentiated cells were washed with PBS and fixed with 10% neutral buffered formalin for 30 min. The cells were washed with distilled water and replaced with 60% isopropanol. The cells were stained with 0.6% (w/v) Oil Red O solution (Oil Red O in isopropanol) for 50 min at room temperature. Stained cells were rinsed twice with distilled water. Photographs were taken by microscope (Nikon Eclipse TS100-F, Japan), the dye was extracted from cells using isopropanol, and absorbance was measured at a wavelength of 540 nm using a Synergy H1 hybrid reader (BioTek, Winooski, VT, USA).

### RNA isolation

Total RNA was isolated from cells using Trizol (Invitrogen, Paisley, UK), according to the manufacturer’s protocol. Chloroform was added, and samples were incubated for 5 min at room temperature. The sample was incubated for 5 min at room temperature and centrifuged at 12,000 × *g* for 15 min at 4 °C. After incubating the sample for 10 min at room temperature, the same volume of isopropanol (Millipore, Bilerica, MA, USA) was added, and samples were centrifuged at 12,000 × *g* for 10 min. After the supernatant was discarded, the pellet was washed with 75% ethanol, centrifuged at 7,500 × *g* for 5 min, and then air-dried. The concentration of RNA was measured using a NanoDrop^TM^ 2000c (Thermo, Bremen, Germany).

### Quantitative real-time PCR

For reverse transcription, 100 ng of total RNA was applied to obtain cDNA using a SuperScript First-Strand Synthesis System for RT-PCR (Invitrogen, cat# 11904-018, Waltham, Massachusetts, USA). qRT-PCR was performed with an applied Biosystems Mx3005P qPCR System (Applied Biosystems, Foster City, CA) using SYBR Green PCR Mastermix reagent (Qiagen, Valencia, CA). Sequences of primers used for PCR are listed in Table 1. Relative mRNA expression levels were compared using the 2^−ΔΔCt^ method.

**Table 1:** List of real time PCR primers and sequences.

Type		Gene	Primer	Primer Sequence
**Adipogenic genes**	**Inhibitor**	***Dlk1***	forward	5′-CGGGAAATTCTGCGAAATAG-3′
			reverse	5′-TGTGCAGGAGCATTCGTACT-3′
	**Early gene**	***Cebpb***	forward	5′-ATCGACTTCAGCCCCTACCT-3′
			reverse	5′-TAGTCGTCGGCGAAGAGG-3′
		***Cebpd***	forward	5′-TTCAACAGCAACCACAAAGC-3′
			reverse	5′-CTAGCGACAGACCCCACAC-3′
	**Key genes**	***Cebpa***	forward	5′-AGCTGCCTGAGAGCTCCTT-3′
			reverse	5′-GACCCGAAACCATCCTCTG-3′
		***Pparg***	forward	5′-TGCTGTTATGGGTGAAACTCTG-3′
			reverse	5′-CTGTGTCAACCATGGTAATTTCT-3′
**Lipogenic genes**		***Fabp4***	forward	5′-CAGCCTTTCTCACCTGGAAGG-3′
			reverse	5′-TTGTGGCAAAGCCCACTC-3′
		***Srebp1***	forward	5′-TCAAGCAGGAGAACCTGACC-3′
			reverse	5′-TCATGCCCTCCATAGACACA-3′
		***Slc2A4***	forward	5′-GACGGACACTCCATCTGTTG-3′
			reverse	5′-GCCACGATGGAGACATAGC-3′
		***Rock1***	forward	5′-TGCTAACCAAAGATATTGAAATGCT-3′
			reverse	5′-TTTATTTCTTCCTCCTTCTTCAATTT-3′
		***Rock2***	forward	5′-CAGTCCCTGGGTAGTTCAGC-3′
			reverse	5′-GCCTGGCATATACTCCATC-3′

### Measurement of mitotic clonal expansion

3T3-L1 cells were cultured on 12-well plates and differentiated through the addition of DMI with or without 5 μM or 10 μM KD025. Cells were trypsinized, and the cell number was counted using microcopy (Nikon Eclipse TS100-F, Japan) on days 0, 2, 3 and 4.

### Transfection of siRNAs

siRNAs were introduced in 3T3-L1 cells by transient transfection with a Lipofectamine^TM^RNAiMAX reagent (Invitrogen). Cells were further incubated for 2 days to reach confluence and then used for the analysis of insulin signaling pathway or DMI treatment. The luciferase reporter control siRNA was purchased from Invitrogen. siRNA for murine ROCK1 and ROCK2 were synthesized as Stealth^TM^ siRNA duplexes (Invitrogen). The sequences used are as follows: ROCK1 S1, 5′-GCACGCCUAACUGACAAGCACCAAU-3′; ROCK1 S2, 5′-UCCAAGUCACAAGCAGACAAGGAUU-3′; ROCK2 S1, 5′-CCGGACCCAUGGAUCAGAGAUAAUU-3′; ROCK2 S2, 5′-GCAGGAAACUCAGAAGCGUUGUCUU-5′.

### Western blot analysis

Cells were suspended in lysis buffer (20 mM tris pH 7.5, 5 mM EDTA, 10 mM Na_4_P_2_O_7_, 100 mM NaF, 2 mM Na_3_VO_4_, 1% NP-40, 1 mM PMSF, 10 μg/mL aprotinin, and 10 μg/mL leupeptin) and 20 μg of each fraction was separated by SDS-PAGE, followed by western blot analysis. The membranes were incubated with antibodies; phospho-Ser^473^Akt, phospho-Thr^308^Akt, phospho-Ser^632/635^IRS-1, phospho-ERM, and ERM from Cell Signaling Technology (Beverly, Massachusetts); Akt, ROCK1 (H-85), ROCK2 (H-85), PPARγ, α-actin, phosphor-cofilin, GAPDH and monoclonal antibodies specific for RhoA (26C4) from Santa Cruz Biotechnology (Dallas, Texas); phospho-Y^612^ IRS-1 (Invitrogen); phospho-Thr^853^ MYPT1 was from CyclexMBL (Japan). The bands were visualized with a ChemiDoc imaging system (Bio-Rad) and quantified by Image Lab software (Bio-Rad) and ImageJ software (National Institutes of Health, ver.1.51k).

### Confocal microscopy

Cells on coverslips were washed with PBS and fixed on ice with 3% paraformaldehyde/PBS for 10 min, and then washed with PBS. Cells were permeabilized with 0.1% triton X-100 in PBS for 3 min, washed with PBS, and incubated in blocking solution (5% milk) for 10 min. Cells were stained for F-actin by Alexa Fluor 448 phalloidin (Invitrogen) and incubated with anti-paxillin antibodies (BioLegend, San Diego, CA), diluted at 1:100 in blocking buffer, overnight at 4 °C. Nuclei were counterstained with 4′,6-diamidino-2-phenylindole (DAPI). Subcellular localization of the proteins was visualized using a Nikon Eclipse Ti inverted microscope (Nikon, Tokyo, Japan) and analyzed using NIS-Elements software (ver. 4.0; Nikon Corporation, Tokyo, Japan).

### Statistical analysis

All experiments were undergone more than three times independently and the representative one was displayed. Data were expressed as means ± SEM or SD. Comparisons between means were performed using unpaired Student’s t-tests.

## Electronic supplementary material

Supplementary data
