# Supplementary material for: Anti-adipogenic effects of KD025 (SLx-2119), a ROCK2-specific inhibitor, in 3T3-L1 cells
Source: Sci Rep. 2018 Feb 6;8:2477. doi: 10.1038/s41598-018-20821-3 (PMC5802830; doi:10.1038/s41598-018-20821-3)
Supplement: Supplementary file 1 — Supplementary data [file 41598_2018_20821_MOESM1_ESM.pdf]

SUPPLEMENTARY DATA

Anti-adipogenic effects of KD025 (SLx-2119), a ROCK2-specific inhibitor, in 3T3-L1 cells.

Duy Trong Vien Diep[1*], Kyungki Hong[1*], Triyeng Khun[1*], Mei Zheng[1], Asad ul-Haq[1], Hee-Sook Jun[1,3,4], Young-Bum Kim[2,3†], and Kwang-Hoon Chun[1†]

[1]Gachon Institute of Pharmaceutical Sciences, College of Pharmacy, Gachon University, Incheon 21936, Republic of Korea

[2]Division of Endocrinology, Diabetes and Metabolism, Beth Israel Deaconess Medical Center and Harvard Medical School, Boston, Massachusetts, United States

[3]Lee Gil Ya Cancer and Diabetes Institute, Gachon University, Incheon 21999, Republic of Korea

[4]Gachon Medical Research Institute, Gil Hospital, Incheon 21565, Republic of Korea

[†]Correspondence to:

Kwang-Hoon Chun, College of Pharmacy, Gachon University, Incheon 21936, Republic of Korea

Tel.: +82-32-820-4951

E-mail: khchun@gachon.ac.kr

Young-Bum Kim, 330 Brookline Ave, CLS-745, Boston, MA 02215, USA

Tel.: +1-617-735-3216, Fax: +1-617-735-3323

E-mail: ykim2@bidmc.harvard.edu

[*]The authors contributed equally

Supplemental Figures

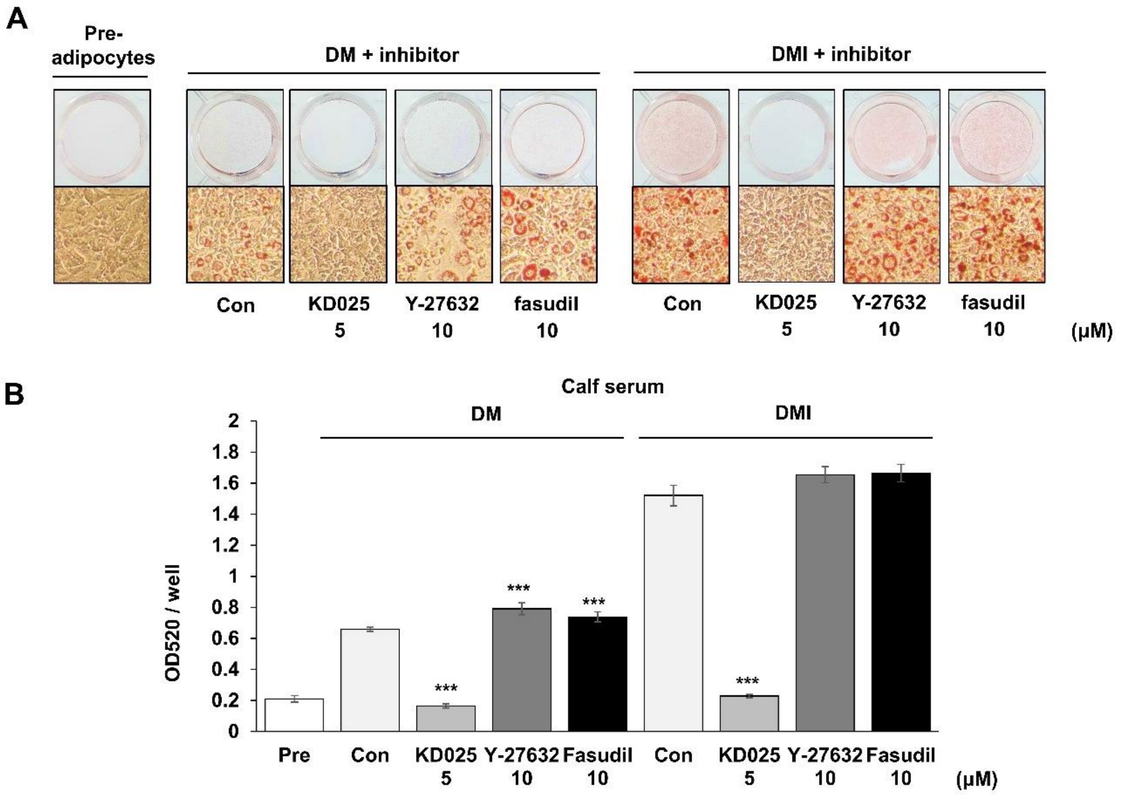

Supplementary Figure S1. Comparison of the effects of ROCK inhibitors during 3T3-L1 adipogenesis. (A) 3T3-L1 pre-adipocytes were cultured in 10% calf serum containing culture media. After 2 days incubation post-confluence, they were differentiated by incubation in DMI with KD025, Y-27632, or fasudil, as indicated, and stained with Oil Red O on day 8. (B) Lipid accumulation was assessed by measuring absorbance at 520 nm of Oil Red O. Data is expressed as means ± S.E. based on triplicate experiments. *, $p < 0.05$; **, $p < 0.01$; ***, $p < 0.001$ *vs.* DM control.

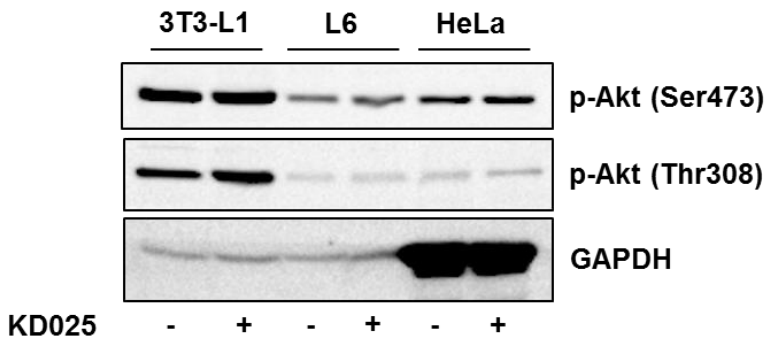

Supplementary Figure S2. Comparison of the effects of ROCK inhibitors in normal growth condition. Sub-confluent 3T3-L1, L6, or HeLa cells were treated with 5 µM of KD025 (in 10% NBCS or FBS/DMEM) for 1 day. Cell lysates were obtained and subjected to SDS-PAGE for immunoblot analysis using anti-phospho-IRS-1 (Tyr608) and anti-phospho-Akt (Ser473 and Thr308) antibodies. GAPDH was used as a loading control.

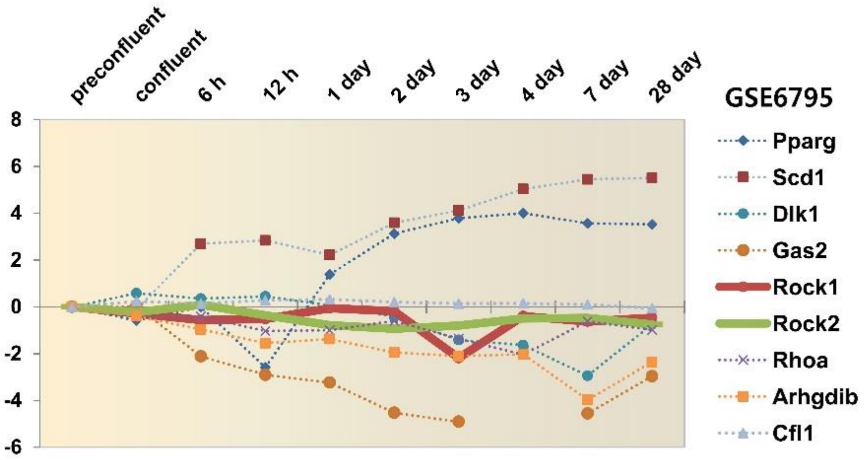

Supplementary Figure S3. ROCK mRNA level during adipogenesis. Gene expression level was analyzed during adipogenesis of 3T3-L1 using data (ID : GSE6795) from Gene Expression Omnibus database (https://www.ncbi.nlm.nih.gov/geo/). Up- or down-regulated genes are displayed for comparison. *Cfl1*, cofilin gene, was consistent in the mRNA level through the differentiation.

Raw Images used in article

Figure 5

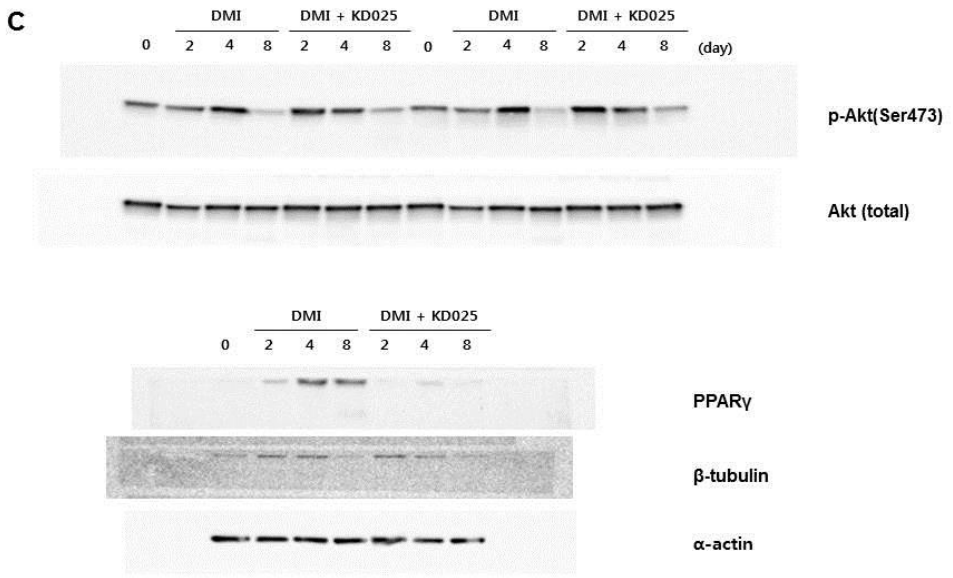

Figure 6

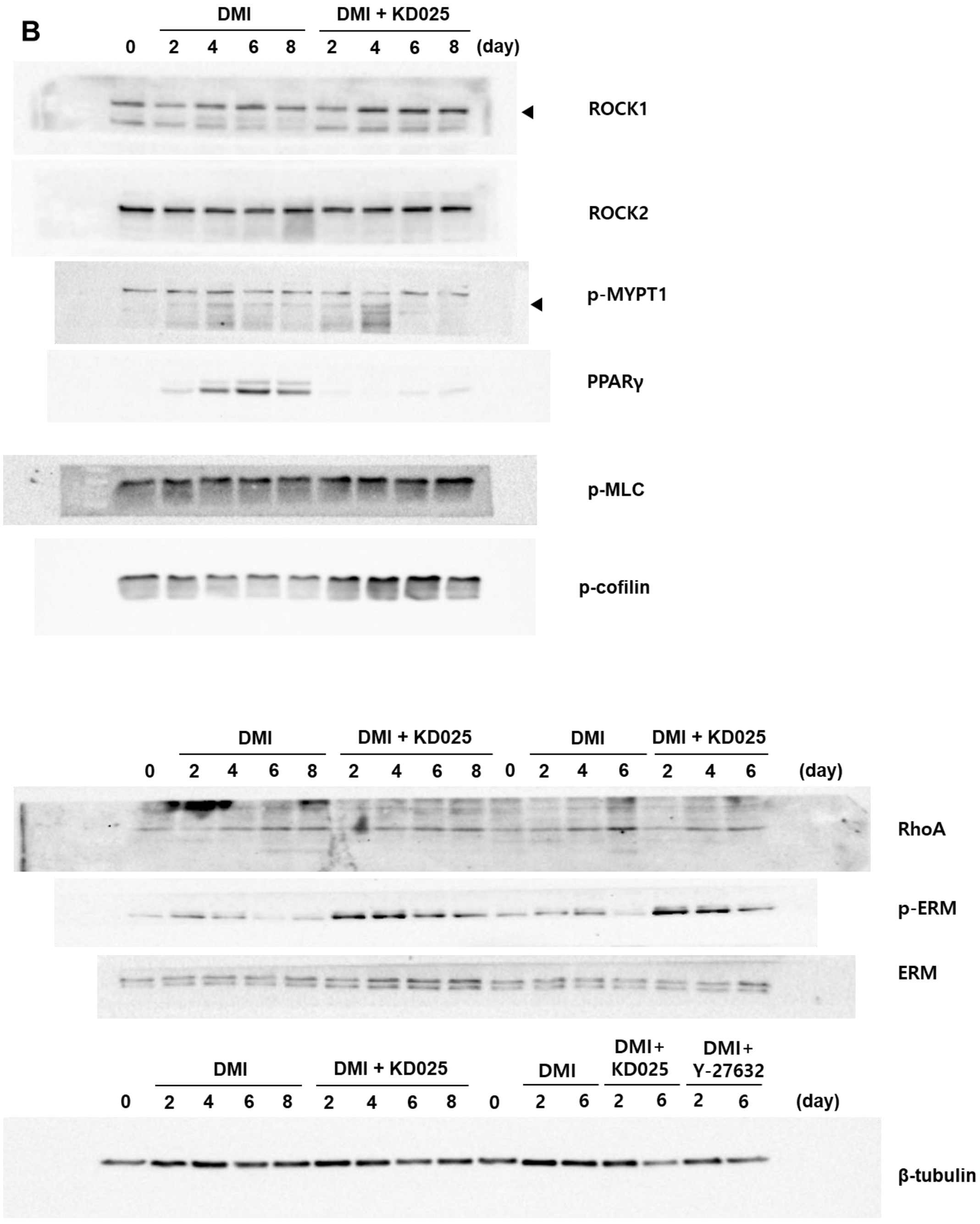
B
DMI
DMI + KD025
0 2 4 6 8 2 4 6 8 (day)
ROCK1
ROCK2
p-MYPT1
PPARγ
p-MLC
p-cofilin
DMI
DMI + KD025
DMI
DMI + KD025
0 2 4 6 8 2 4 6 8 0 2 4 6 2 4 6 (day)
RhoA
p-ERM
ERM
DMI
DMI + KD025
DMI
DMI+ KD025
DMI+ Y-27632
0 2 4 6 8 2 4 6 8 0 2 6 2 6 2 6 (day)
β-tubulin

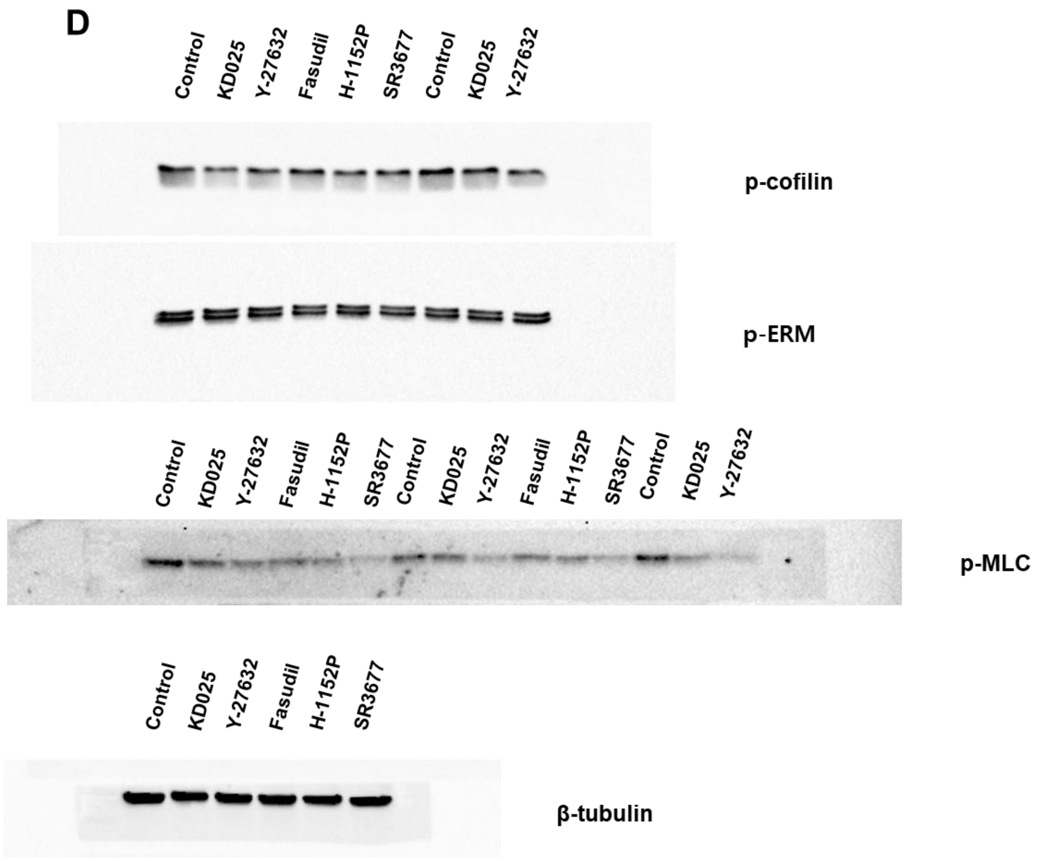
D
Control
KD025
Y-27632
Fasudil
H-1152P
SR3677
Control
KD025
Y-27632
p-cofilin
p-ERM
Control
KD025
Y-27632
Fasudil
H-1152P
SR3677
Control
KD025
Y-27632
Fasudil
H-1152P
SR3677
Control
KD025
Y-27632
p-MLC
Control
KD025
Y-27632
Fasudil
H-1152P
SR3677
β-tubulin

Figure 7

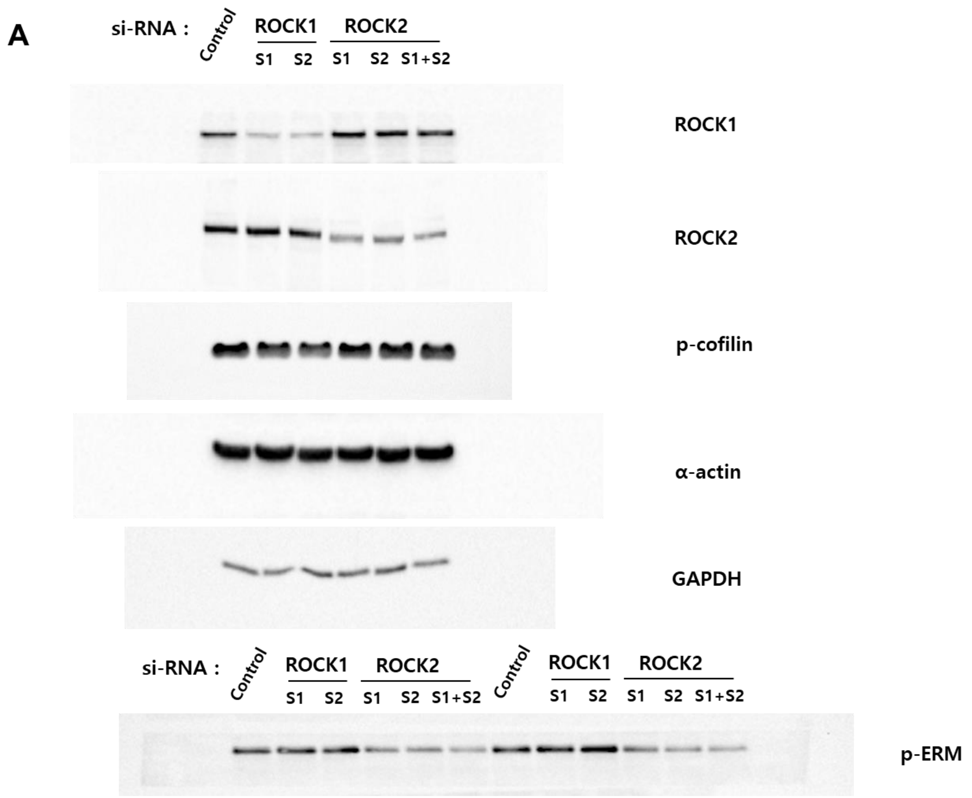
A
si-RNA :
Control
ROCK1
ROCK2
S1
S2
S1
S2
S1+S2
ROCK1
ROCK2
p-cofilin
α-actin
GAPDH
si-RNA :
Control
ROCK1
ROCK2
S1
S2
S1
S2
S1+S2
Control
ROCK1
ROCK2
S1
S2
S1
S2
S1+S2
p-ERM
